# SAN ANTONIO TRANSPORTATION PLANNING SYSTEM: MAPPING INFRASTRUCTURE CAPACITY AND COORDINATION

**DOI:** 10.1093/geroni/igad104.0710

**Published:** 2023-12-21

**Authors:** Laura Keyes, Sowmya Balachandran, Simon Andrew

**Affiliations:** University of North Texas, Denton, Texas, United States; University of North Texas, Denton, Texas, United States; University of North Texas, Denton, Texas, United States

## Abstract

Ride Connect Texas and its partnering agencies seek to develop an expanded senior mobility management system supported by a One Call One Click transportation planning system. Its purpose is to enhance on-demand transportation system coordination and the application of technology, providing consumers, service providers, and mobility managers with direct access to comprehensive transportation information. GIS mapping is used to help develop a spatial awareness of the transportation challenges among older people and individuals with a disability and the current system capacity. Interviews with transit, non-transit, and health and human service agencies will reveal factors that influence coordination among providers and opportunities for capacity expansion. Surveys and focus groups of older adults and individuals with disability will show differentiating characteristics of riders and transportation needs and challenges. GIS mapping will allow us to geocode these findings relative to the user’s street address and zip code, illustrating variations in specialized client needs, service availability, travel behavior, and available amenities for basic daily living. Mapping will identify gaps in transportation services, hot zones revealing high user demand and low system capacity, and the ability to reduce fragmentation of service through seamless technology-based coordination. The mapping element informs the measurement of a community’s transportation system capacity for this user group. The value proposition of this tool is its ability to help any community measure its readiness toward developing a comprehensive implementation strategy for coordinated demand response transportation, including resource mobilization strategies, capacity augmentation, phasing of the system, and implementation timeline.

